# Supplementary material for: GC-ToF-MS Profiling and In Vitro Inhibitory Effects of Selected South African Plants against Important Mycotoxigenic Phytopathogens
Source: Life (Basel). 2023 Jul 30;13(8):1660. doi: 10.3390/life13081660 (PMC10455341; doi:10.3390/life13081660)
Supplement: Supplementary file 1 [file life-13-01660-s001.zip › life-2520875-supplementary.pdf]

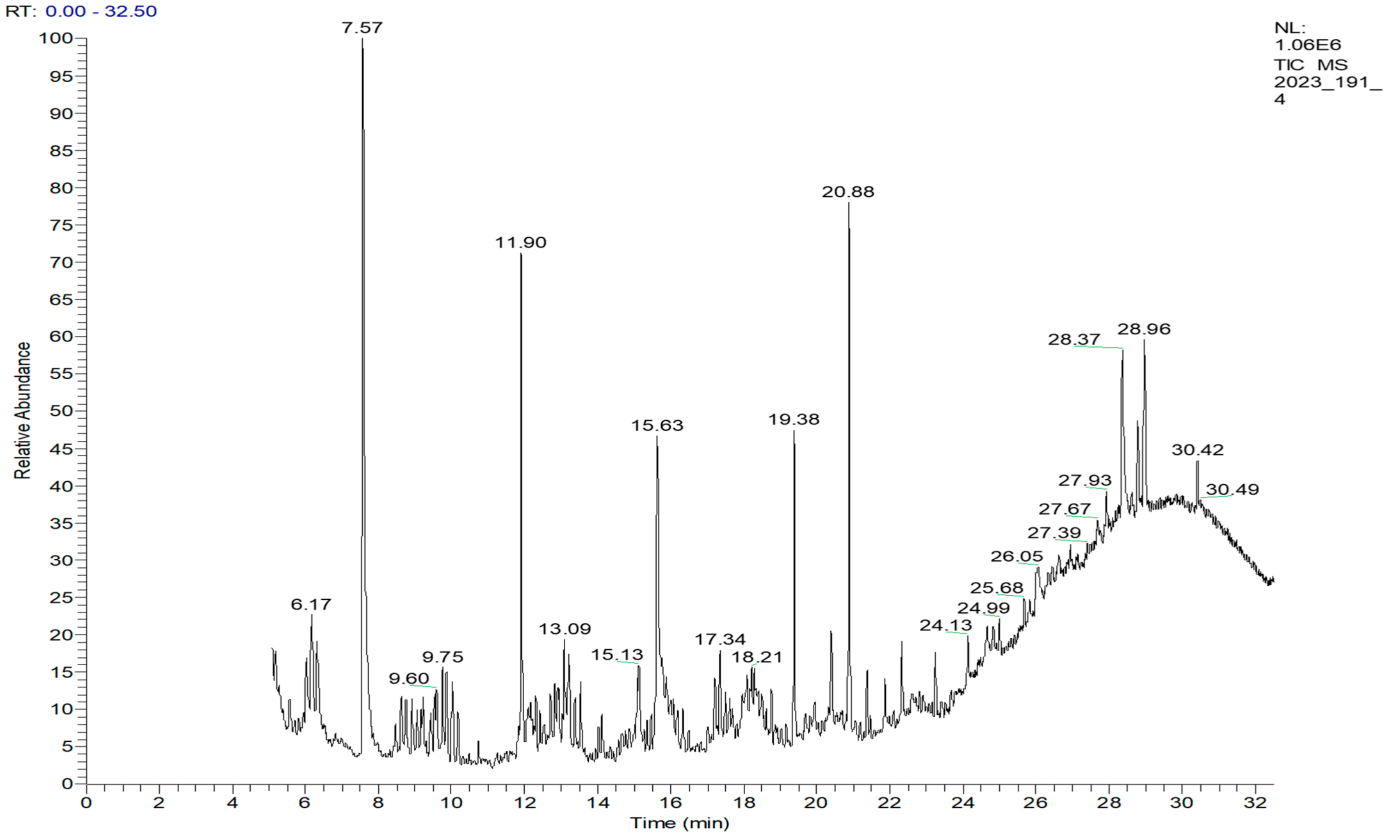

Figure S1. GC-ToF-MS chromatogram of crude organic extract of *Bauhinia galpinii.*

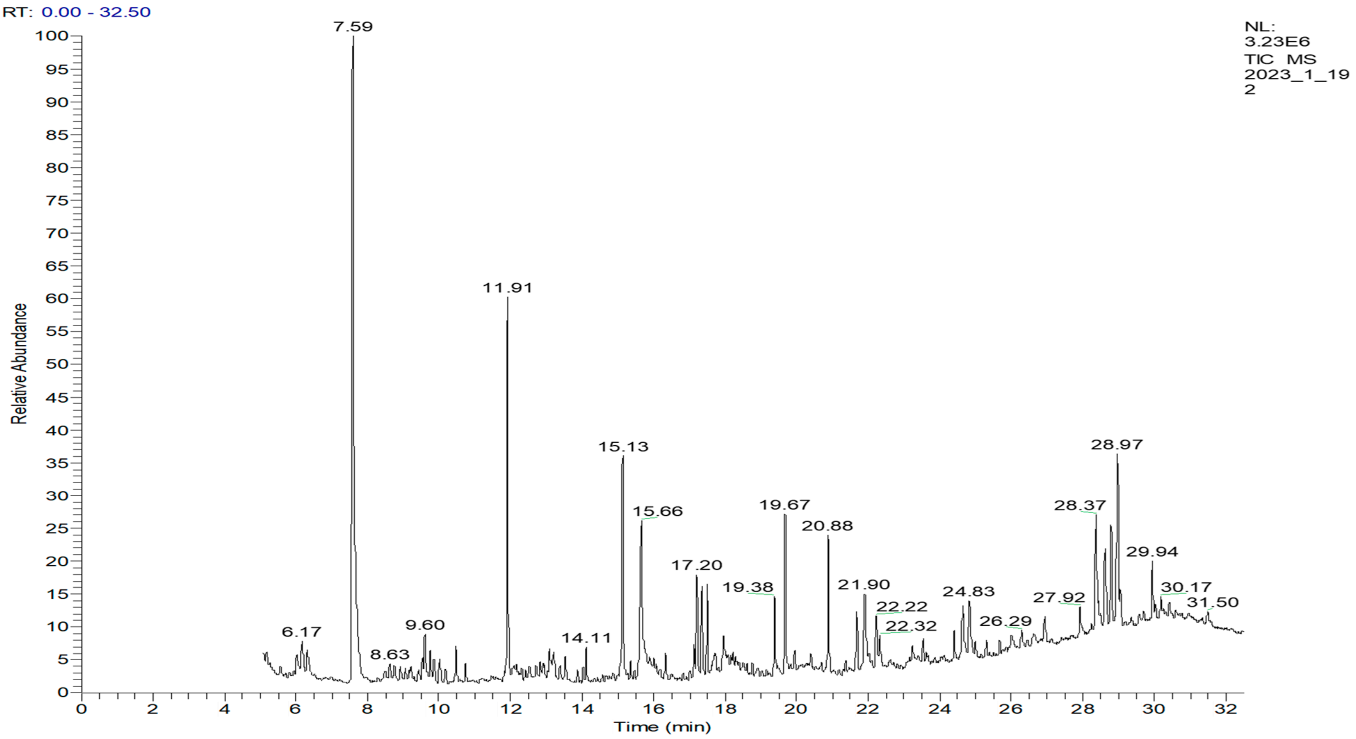

Figure S2. GC-ToF-MS chromatogram of crude organic extract of *Combretum caffrum.*

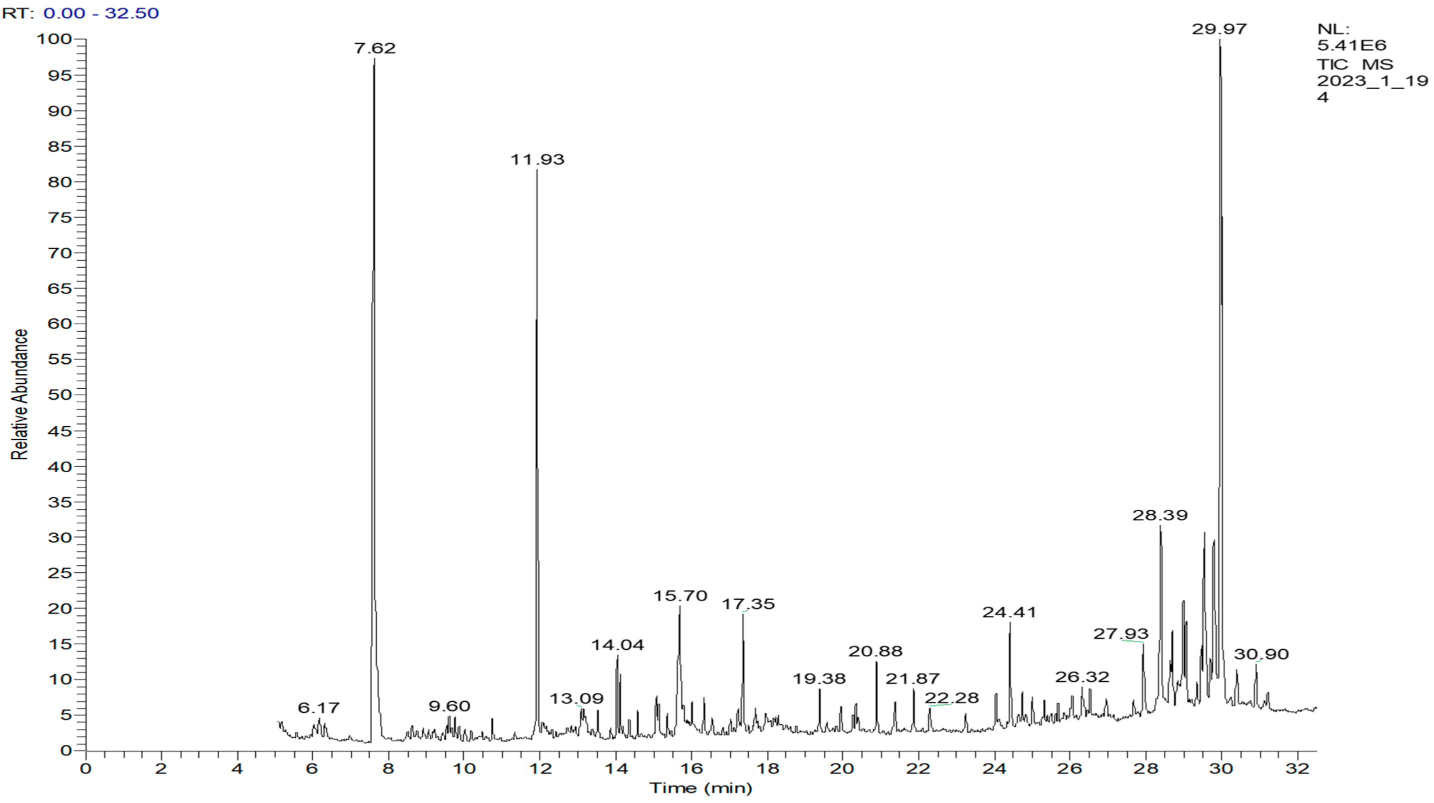

Figure S3. GC-ToF-MS chromatogram of crude organic extract of *Maytenus undata.*

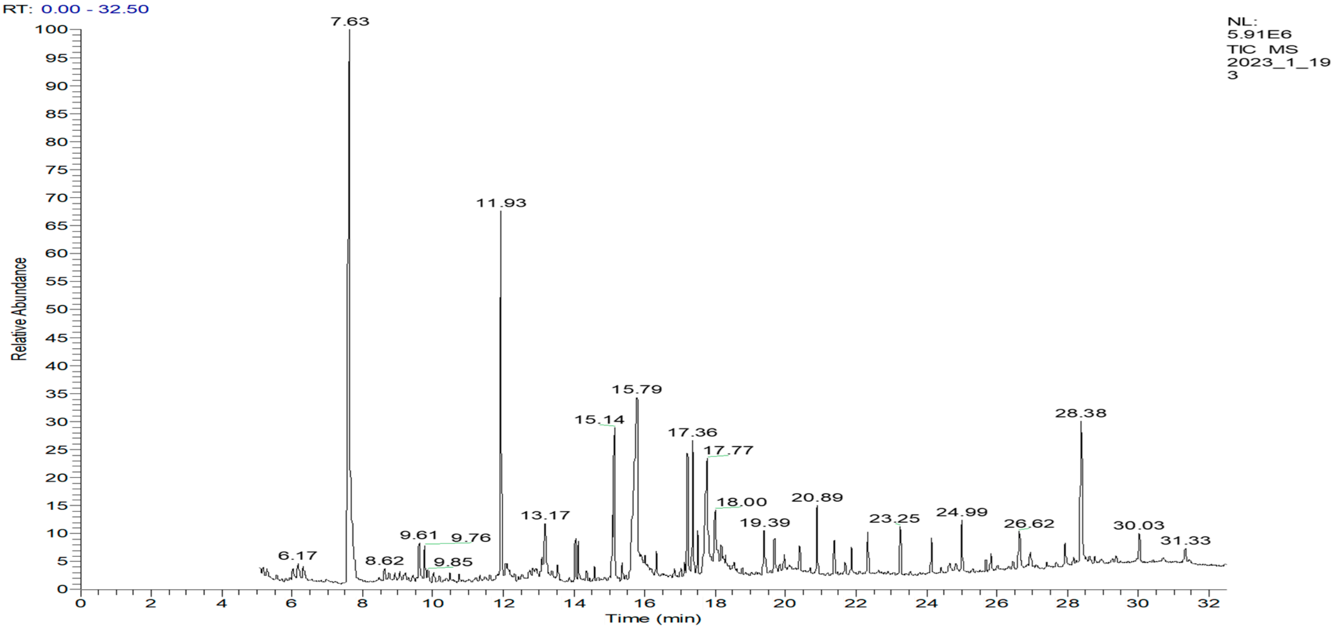

Figure S4. GC-MS chromatogram of crude organic extract of *Markhamia obstufolia.*
